# Arthroscopic reduction with non-fixation for Broberg & Morrey type II radial head fracture with mechanical rotation block: a propensity score-matched case-control study

**DOI:** 10.3389/fsurg.2025.1680368

**Published:** 2025-09-16

**Authors:** Yanmao Wang, Shiyang Yu, Jian Ding, Shengdi Lu

**Affiliations:** Department of Orthopedics, Shanghai Sixth People’s Hospital Affiliated to Shanghai Jiao Tong University School of Medicine, Shanghai, China

**Keywords:** radial head fracture, arthroscopic reduction, mechanical block, broberg & morrey classification, randomized controlled trials

## Abstract

**Aims:**

To evaluate arthroscopic reduction without fixation (ARnF) for Broberg & Morrey Type II radial head fractures presenting with a mechanical block to forearm rotation.

**Methods:**

We reviewed 11 patients with Broberg & Morrey Type II radial head fractures and a mechanical rotation block treated with ARnF. Clinical outcomes included elbow range of motion (ROM), the American Shoulder and Elbow Surgeons (ASES) score, and the Disabilities of the Arm, Shoulder and Hand (DASH) score. Outcomes were compared with those of patients who underwent arthroscopic reduction and internal fixation (ARIF) using cannulated screws at 6 weeks, 12 weeks, and 1 year postoperatively.

**Results:**

Eleven patients were included (7 male; mean age 33.6 years). All showed significant postoperative improvement. At each follow-up, mean ROM and both functional scores (ASES and DASH) in the ARnF group were comparable to those in the ARIF cohort.

**Conclusion:**

or Broberg & Morrey Type II radial head fractures with a mechanical rotation block, ARnF achieved outcomes comparable to ARIF while avoiding implant costs and implant-related risks.

**Clinical Trial Registration**: https://www.chictr.org.cn/, identifier (ChiCTR2000035958).

**Level of Evidence:** LEVEL III; Treatment study; Case control study.

## Introduction

When assessing radial head and neck fractures, a number of parameters need to be considered in order to determine treatment (1–3). These include fracture stability, displacement, the extent of joint involvement and the presence of associated complex injuries (2). In radial head fractures, operative indication for fracture instability and displacement are not synonymous (1). The majority of isolated fractures involving only part of the radial head are inherently stable, even when displaced >2 mm. Currently, fracture fragment displacement of >2 mm is often used as a criterion for considering operative treatment (1–3). However, this amount of displacement may be seen in the context of a stable fracture and preserved elbow and forearm motion. Furthermore, long-term follow-up studies have shown successful outcomes with non-operative treatment when forearm motion is preserved (2, 3). In clinical practice, we have rarely seen type 1 fractures become displaced, even with full range of motion. The main indications for radial head surgery are concerns about elbow function and potential complications. According to a previous report, Broberg & Morrey type II radial head fractures were defined as reconstructable radial head fractures with the presence of a mechanical block preventing motion (2, 4).

Arthroscopy is increasingly being used in elbow injuries. Arthroscopy improves visualization of the articular surface of the radial head, which leads to a better understanding of the morphology of fracture lines and fragments and offers the possibility of clear testing of joint stability (5, 6). It also allows the accurate assessment and treatment of associated intra-articular pathology, such as traumatic cartilage lesions, ligament injuries and loose bodies. Another advantage when compared with open reduction and internal fixation (ORIF) is the limited soft tissue dissection, which can improve healing by maintaining blood supply, may decrease the analgesic requirement and might shorten the hospital stay (6).

We hypothesized that Broberg & Morrey type II radial head fractures with mechanical rotation block can be converted to stable Broberg & Morrey type I radial head fractures by arthroscopic reduction and holding by intact annular ligaments instead of extra implantation. This study aimed to evaluate the clinical effects of our technique of using ARnF for Broberg & Morrey Type II radial head fractures with mechanical rotation block. We described our treatment algorithm for this type of injury.

## Materials and methods

### Study participants

We conducted a retrospective, consecutive, single-surgeon case series with prospectively collected data for patients who were treated ARnF for Broberg & Morrey Type II radial head fractures with mechanical rotation block in Shanghai Jiao Tong University affiliated Sixth People's Hospital between January 2019 and April 2022. Surgical treatment was indicated to all patients after comprehensive history, physical examination and computed tomography (CT) showed Broberg & Morrey Type II radial head fractures with mechanical rotation block.

The definition of Broberg & Morrey Type II radial head fractures with mechanical rotation block in the present study was formulated based on Broberg & Morrey classification (7) as follows: a. fracture of the radial head or/and radial neck displaced >2 mm and involving ≥30% of the joint surface confirmed by computed tomography (CT); b. close fracture; c. split radial head with partially continuous epiphysis; d. forearm rotation limitation (less than 50° supination or pronation) is present on physical examination under hematoma anesthesia to <50° of supination or pronation on physical examination, even after a hematoma block (to differentiate pain from mechanical restriction), indicating a true mechanical block; we selected the 50° threshold because this value corresponds to the minimum functional forearm rotation arc required for daily activities (8).

A total of 14 Broberg & Morrey Type II radial head fractures with mechanical rotation block patients were identified. After exclusion of patients with incomplete data, the present study included 11 patients. No data imputation was performed for missing values; only patients with complete data were included in the analysis. The study and the analysis plan were approved by the Institutional Review Board (Research Ethics Committee) of the Shanghai Sixth People's Hospital affiliated to Shanghai Jiao Tong University School of Medicine (IRB number: 2020-KY-037(K)). The study was registered in the Chinese Clinical Trial Registry (Number: ChiCTR2000035958). Informed consent was obtained by all participants to publish the information/images in an online open access publication. All methods were performed in accordance with the relevant guidelines and regulations.

### Surgical techniques, postoperative interventions and rehabilitation

Elbow arthroscopy was performed using a standardized technique as described (5, 6). The patient is placed in the prone position under general anesthesia. The arm is suspended over a padded arm board with the elbow flexed at 90 degrees and the forearm hanging freely. The elbow is initially insufflated with saline through the soft tissue. The first anteromedial portal is used for visualization. An anterolateral portal is used for instrumentation. The hemarthrosis and debris are washed out. The joint is inspected and associated lesions are sought. Pronation and supination allow a more extensive view of the radial head and fracture fragments. Usually, we can insert the probe into the fracture line and use repeated lever action to release the displaced fragment. Then restore the height of each piece of fragment, push them all together in case of formation Protruding edge remove all the small unreducible osteochondral fragment. After assessing the quality of the fracture reduction, pronation and supination are performed to check if there is still block or not. Reduced fragments should remain in the appropriate position if the elbow joint is sufficiently stable.

A sling was used for one week after operation the operation, and continuous passive motion (CPM) was used initiated on the first postoperative day. CPM sessions (∼20 min, 3–4 times daily) were applied during the first postoperative week to gently mobilize the elbow through a pain-free flexion-extension arc. Early mobilisation of extension, flexion, and pronation or supination forearm rotation was initiated on in the second postoperative week under the supervision of an orthopaedist or a physical therapist. Patients were instructed to perform these exercises at least three times daily as tolerated, gradually increasing their range of motion over time. Unrestricted shoulder and wrist motion was encouraged. Shoulder and wrist movements were unrestricted throughout the rehabilitation. Indomethacin (25 mg, administered orally three times per day) was prescribed for 3 weeks to prevent heterotopic ossification. All patients received parecoxib (40 mg twice daily) to relieve pain and allow for early active elbow exercises in the first 2 weeks.

### Blinding

Three independent orthopedic surgeons (J.D., Y.W., and S.L.), each with experience in over 50 cases of arthroscopic treatment for elbow trauma or myotendinous disorders, were engaged in this study. One of the three orthopedic surgeons participating in the study was exclusively responsible for performing ARnF procedures and did not participate in any ARIF surgeries. All ARIF surgeries conducted during the study were performed by the other two surgeons.

The assessors who measured the primary outcomes (range of motion and forearm rotation) were blinded to group assignments, and all measurements were performed in a standardized manner using a goniometer. To mitigate the potential for bias, the assessors were not involved in patient care and were instructed to avoid examining the surgical incisions directly.

### Baseline measurements

All patients' data were extracted from the Elbow injury database in Shanghai Jiao Tong University affiliated Sixth People's Hospital. The Elbow injury database is one of the disease-specific databases which established in January 2018. Data for the present study included data of birth, sex, body mass index (BMI), time to surgery (h), mechanism of injury, whether dominant hand is involved, Mason classification, Broberg & Morrey classification, suspected pathology based on imaging. The technique of using ARnF for Broberg & Morrey Type II radial head fractures with mechanical rotation block was started in January 2019 for the first case. The control group was defined as the patients with Broberg & Morrey type II radial head fractures with mechanical rotation block who were treated with arthroscopic reduction and internal fixation (ARIF) with cannulated screw.

### Propensity score matching

Patients included in this study should have follow-up period no less than 1 year after discharge of the hospital. Before the propensity score matching, patients with Broberg & Morrey type II radial head fractures with mechanical rotation block who were treated with ARIF with cannulated screw was first extracted. Nearest neighbor matching was used as the matching algorithm. Caliper was set at 0.01 level. A propensity score was thus generated by a logistic regression model. Covariates for matching included age, sex, BMI, education level, type of insurance, time to surgery (h), mechanism of injury, whether dominant hand is involved, Mason classification, Broberg & Morrey classification, suspected pathology based on imaging. Based on the propensity score, patients treated with ARnF were matched 1:1 with patients treated with ARIF.

Finally, 11 Broberg & Morrey Type II radial head fractures with mechanical rotation block patients treated with ARnF were matched 11 Broberg & Morrey Type II radial head fractures with mechanical rotation block patients who treated with ARIF. The baseline characteristics were well matched (Table 1).

**Table 1:** Characteristics of patients treated with arthroscopic reduction with non-fixation.

Variable	P1	P2	P3	P4	P5	P6	P7	P8	P9	P10	P11
Gender	Male	Female	Male	Male	Female	Male	Male	Female	Female	Male	Male
Age	33	23	41	36	37	31	36	33	38	33	28
BMI (kg/m^2^)	23	30	30	23	23	24	20	27	28	25	20
Education level	<High School	<High School	≥High School	<High School	≥High School	≥High School	≥High School	≥High School	≥High School	≥High School	<High School
Type of insurance	Government	Commercial	Government	Self-financed	Government	Government	Government	Government	Commercial	Government	Government
Time to surgery (Days)	4	6	3	5	12	4	12	3	2	4	13
Mechanism of injury	Fall	Fall	Car Accident	Fall	Fall	Car Accident	Fall	Fall	Fall	Car Accident	Fall
Dominant hand involved	Yes	No	Yes	Yes	No	Yes	Yes	Yes	Yes	No	No
Mason classification	2	2	2	2	2	2	2	2	2	2	2
Broberg & Morrey classification	II	II	II	II	II	II	II	II	II	II	II
Position of forearm rotation block*	31	−25	10	42	30	−14	25	20	−37	5	45
Suspected pathology based on imaging**	MCL injury	None	None	None	None	None	None	None	MCL injury	None	None

* +: at pronation; −: at supination.

** MCL: medial collateral ligament.

### Outcome measures

Patients' outcome data at day of discharge (E0), 6 weeks (E1), 12 weeks (E2), and 1 year (E3) after discharge was extracted, including ROM of flexion and extension, ROM of forearm rotation, ASES elbow function subscale, ASES elbow pain subscale and DASH score.

The ROM of flexion and extension and ROM of forearm rotation were used as the primary outcome of this study.

### Statistical analysis

No formal power calculation was performed, given the retrospective design and limited sample size of this study; we included all eligible patients from the study period. To account for rounding in range-of-motion measurements, we applied a *p*-value correction method following Zdravkovic and Jost's recommendation, setting the significance threshold at 0.026 for outcomes assessing mean differences in range of motion (9). For other outcomes, a *p*-value of 0.05 was considered significant.

For the primary outcome analysis, we employed a linear mixed model for repeated measures, considering the correlation among range-of-motion measurements within the same patient and adjusting for baseline range of motion and patient treatment preferences. Similar statistical analyses were applied to continuous secondary outcomes. Dichotomous secondary outcomes were compared between groups using Cochran-Mantel-Haenszel chi-square tests, while controlling for baseline contracture severity.

To manage multiple endpoints, we assessed secondary endpoints hierarchically. If an endpoint didn't reach significance, no further conclusions were drawn for lower-ranked endpoints. Similarly, exploratory analyses were not adjusted for multiplicity. Statistical analyses were performed with R (version 4.3.3).

## Results

### Patients

Detailed characteristics, baseline, and postoperative clinical results of patients treated with ARnF were listed in Tables 1, 2. The average age at the time of surgery was 33.6 (range, 23–41 years). The dominant arm was affected in 63.6% patients (7/11 patients). Majority of patients resulted from fall (7/11 patients). The positions of forearm rotation block were also noted. 2 patients were suspected of medial collateral ligament injury based on preoperative imaging.

**Table 2:** Preoperative and postoperative clinical results of patients with ARnF.

Outcome*	Time point	P1	P2	P3	P4	P5	P6	P7	P8	P9	P10	P11
Range of motion (°)	Postop	129	120	125	130	128	130	120	120	130	125	130
	Postop 6 weeks	127	116	127	103	122	122	112	116	128	109	113
	Postop 12 weeks	128	134	131	130	132	132	116	117	140	125	138
	Postop 1 year	127	133	132	128	135	142	119	115	140	128	139
Range of forearm rotation(°)	Postop	160	155	150	155	150	160	155	160	160	155	155
	Postop 6 weeks	160	158	153	161	164	164	152	162	162	160	156
	Postop 12 weeks	169	172	160	164	173	172	162	172	161	169	170
	Postop 1 year	172	170	163	164	180	177	163	174	163	174	178
ASES elbow function subscale (points)	Postop	N/A	N/A	N/A	N/A	N/A	N/A	N/A	N/A	N/A	N/A	N/A
	Postop 6 weeks	79	81	87	82	82	80	79	89	81	85	87
	Postop 12 weeks	90	90	92	100	98	87	91	89	99	85	98
	Postop 1 year	94	92	93	100	98	88	94	92	99	91	98
ASES elbow pain subscale (points)	Postop	N/A	N/A	N/A	N/A	N/A	N/A	N/A	N/A	N/A	N/A	N/A
	Postop 6 weeks	3	1	2	1	1	1	1	2	1	1	2
	Postop 12 weeks	1	0	0	0	0	1	0	1	0	0	0
	Postop 1 year	0	0	0	0	0	0	0	0	0	0	0
DASH score (points)	Postop	N/A	N/A	N/A	N/A	N/A	N/A	N/A	N/A	N/A	N/A	N/A
	Postop 6 weeks	20	22	12	14	26	15	23	11	24	12	11
	Postop 12 weeks	7	4	7	0	8	10	5	13	5	12	6
	Postop 1 year	8	3	10	0	2	4	4	7	6	10	8
Hospital stays (days)		3	3	3	3	3	3	3	3	3	3	3
Times of fluoroscope		1	1	1	2	1	1	2	1	1	1	2
Operation time (min)		40	44	44	43	40	48	39	46	37	42	35

* N/A, not applicable; ARnF, arthroscopic reduction with non-fixation; ASES, American shoulder and elbow score; DASH, disability of the arm, shoulder and hand.

### Primary outcomes

Patients received ARnF was similar to patients received ARIF with regard to the rate of recovery as well as the final improvement in range of motion (Table 3). At the 12-month follow-up, a treatment difference of 0.3° in range of motion was observed (*p* = 0.92). A treatment difference of 3.6° in range of forearm rotation was also observed (*p* = 0.42) (Table 3). The typical case data of patient No. 8 are presented in the Supplementary Material.

**Table 3:** Primary and key secondary outcomes.

Outcome*	ARnF (*n* = 11)	ARIF (*n* = 11)	*p* Value
Primary outcome			
Range of motion at 1 yr (deg)	130.4 ± 6.7	130.7 ± 8.5	0.92
Range of forearm rotation at 1 yr(deg)	173.1 ± 5.1	170.7 ± 6.5	0.42
Secondary outcomes			
Range of motion at 6 wk (deg)	114.3 ± 10.9	117.7 ± 8.2	0.27
Range of motion at 12 wk (deg)	126.6 ± 5.2	129.4 ± 7.6	0.33
Range of forearm rotation at 6 wk (deg)	159.1 ± 6.7	159.3 ± 4.1	0.94
Range of forearm rotation at 12 wk (deg)	169.9 ± 5.2	167.6 ± 4.9	0.37
Percentage of lost motion recovered at 1 yr	43.4% ± 6.6%	43.3% ± 8.7%	0.94
Percentage of lost rotation recovered at 1 yr	56.5% ± 19.0%	57.8% ± 20.4%	0.88
ASES elbow function subscore (points)			
6 wk	84.4 ± 4.4	82.9 ± 3.5	0.53
12 wk	90.7 ± 3.3	92.5 ± 5.2	0.25
1 yr	93.5 ± 4.1	94.5 ± 3.8	0.54
ASES elbow pain subscore (points)			
6 wk	1.5 ± 0.7	2.4 ± 0.7	<0.05
12 wk	0.3 ± 0.5	0.3 ± 0.5	1.00
1 yr	0	0	N/A
DASH score (points)			
6 wk	16.9 ± 5.9	17.3 ± 5.8	0.91
12 wk	7.7 ± 6.8	7.0 ± 3.7	0.60
1 yr	6.0 ± 5.7	5.6 ± 3.3	0.86

DASH, Disability of the arm, shoulder and hand score; ASES, American shoulder and elbow score; yr, year; wk, week; deg, degree.

* Continuous variables were presented as mean ± standard deviation.

### Secondary outcomes

The range of motion, range of forearm rotation, and Patient-reported outcome measures (PROMs) scores were comparable between both groups across all time points, except for a notable variation in the ASES pain subscale favoring the new technique group at the 6-week mark (as shown in Table 3). For tourniquet time, patients treated with ARnF showed significant less minutes compared to patients treated with ARIF (*p* < 0.001) (Table 4). Patients treated with ARnF noted similar times fluoroscope compared to patients treated with ARIF (*p* = 0.89) (Table 4).

**Table 4:** Demographic and baseline characteristics of the patients and operative data.

Characteristic	ARnF (*n* = 11)	ARIF (*n* = 11)	*p* Value
Age (year)			0.06
Mean and standard deviation	33.6 ± 5.0	38.4 ± 9.3	
Range	23–41	28–59	
Gender (no. of patients, %)			1.00
Male	7 (63.6%)	7 (63.6%)	
Education level (no. of patients, %)			1.00
High school and college	7 (63.6%)	8 (72.7%)	
Insurance type (no. of patients, %)			0.82
Government	8 (72.7%)	9 (81.8%)	
Commercial	2 (18.2%)	1 (9.1%)	
Self-financed	1 (9.1%)	1 (9.1%)	
BMI (kg/m^2^)			0.94
Mean and standard deviation	24.8 ± 3.5	24.3 ± 3.6	
Time to surgery (days)			0.68
Mean and standard deviation	6.2 ± 4.0	6.8 ± 2.8	
Mechanism of injury (no. of patients, %)			1.00
Fall	8 (72.7%)	9 (81.8%)	
Car/motorcycle/bicycle accident	3 (27.3%)	2 (18.2%)	
Dominant hand involved (no. of patients, %)	7 (63.6%)	8 (72.7%)	1.00
Mason classification			1.00
Type II	11 (100%)	11 (100%)	
Other type	0	0	
Tourniquet time (min)			<0.001
Mean and standard deviation	35.6 ± 3.9	56.2 ± 8.0	
Range	30–43	47–74	
Times of fluoroscope	1.3 ± 0.5	2.0 ± 0.5	0.89

BMI, body mass index.

### Adverse events

No adverse events were noted in both groups (Table 5).

**Table 5:** Complications and adverse events.

Complications and adverse events	ARnF (*n* = 11)	ARIF (*n* = 11)
No. of patients with at least 1 event	0	0
No. of events (event rate)	0	0
Delayed-onset ulnar neuritis	0	0
No further surgery performed	0	0
Further surgery performed	0	0
Other neuritis	0	0
Elbow stiffness	0	0
No further surgery performed	0	0
Further surgery performed	0	0
Persistent intra-articular pain requiring corticosteroid injection	0	0

### Case example

**Figure 1:**
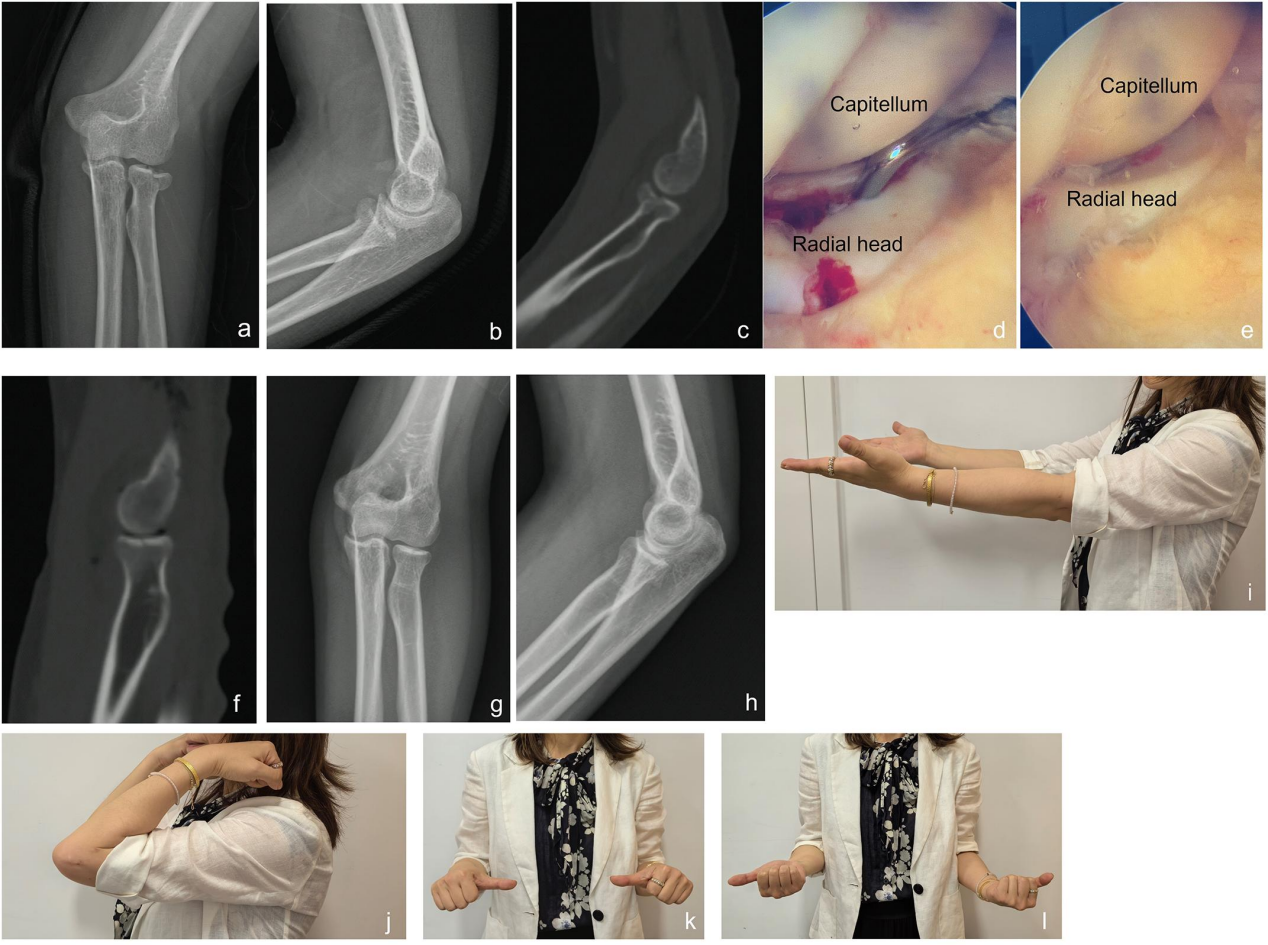
A case example for a patient treated with arthroscopic reduction with non-fixation. A 38-year-old woman (Patient 8) experienced a left radial head fracture due to an accidental fall. **(a,b)** X-ray images after injury; **(c)** CT-scan image after injury; **(d)** intraoperative arthroscopic image showed fragment of radial head; **(e)**: intraoperative arthroscopic image showed that the fragment was stable after reduction; **(f)** postoperative CT-scan image; **(g,h)** x-ray images at 6 months follow-up; **(i–l)** range of motion at 6 months follow-up.

A 38-year-old woman (Patient 8) experienced a left radial head fracture due to an accidental fall. Diagnostic imaging, including x-ray and CT scan, revealed a Mason type II radial head fracture. Physical examination indicated a mechanical block at 20 degrees of pronation. On the third day following the injury, she underwent arthroscopic reduction and non-fixation. Intraoperative arthroscopic imaging confirmed that the fragment was stable after reduction. Follow-up assessments demonstrated satisfactory functional outcomes and nearly normal range of motion (Figure 1).

## Discussion

In this study, we reported on a series of 11 patients who sustained Broberg & Morrey Type II radial head fractures with mechanical rotation block and treated with arthroscopic reduction and non-fixation. And in this analysis using data from a real-world disease-specific database, we demonstrated the noninferiority of using ARnF with Broberg & Morrey Type II radial head fractures with mechanical rotation block when compared to patients using traditional ARIF. In this matched analysis, we found that ARnF yielded outcomes similar to ARIF. The results of the analyses of patients-reported outcome measures also suggest clinical equivalence of ARnF. Likewise, patient-reported outcome measures indicated no clinically significant differences between the ARnF and ARIF groups.

Arthroscopic treatment of elbow pathology is increasing over time; treatments include reduction and fixation of radial head fractures that include associated injuries, such as coronoid fracture or collateral ligament avulsion (10–12). This technique offers multiple advantages: a complete view of the articular surfaces of radial head and coronoid is possible (13), as well as removal of small intra-articular fragments and treatment of trochlear chondral damages or small coronoid fractures that would otherwise require extensive open medial access. There is much less tissue damage with ARIF compared to ORIF; however, ARIF still remains a technically demanding surgery that requires a long learning curve and high technical skills. Complication rate is reported with a huge variability depending on the surgeon's abilities (14). In our series, all ARnF surgeries were performed by a single, experienced surgeon (with >50 prior elbow arthroscopy cases), which likely minimized any learning curve effect. We did not observe any complications or inferior results in the initial cases vs. later cases. Nonetheless, ARnF is indeed a demanding technique, and surgeon proficiency is an important consideration for successful outcomes.

The main indications for ARIF in the radial head are Mason type II fractures of the radial head in which the articular surface is partially involved but the epiphyseal part is intact (6, 15, 16). Mechanical rotation block is a major indicator of either ORIF or ARIF of radial head fracture (17). Recent studies confirmed satisfied clinical outcomes of using ARIF to treat Mason type II radial head fractures (13, 15, 16). In our previous clinical practice, we have found that displaced fragments of Broberg & Morrey type II radial head fractures, provided that lateral collateral ligament (LCL) and annular ligament (AL) were maintained were uninjured, can be stable at the site between the annular ligament and the articular surface of the capitellum after reduction under arthroscopy. Thus, we hypothesize that Broberg & Morrey type II radial head fractures with mechanical rotation block can be converted to stable Broberg & Morrey type I radial head fractures by arthroscopic reduction and non-fixation (intact LCL and AL). The results of this study confirmed our hypothesis based on the equivalence of functional outcome and the incidence of complication between ARnF and ARIF. In addition to the 11 patients in this study who had no fracture fragment displacement at follow-up, there were 3 other patients who also underwent ARnF for Broberg & Morrey type II radial head fractures with mechanical rotation block at our institution, and these 3 patients also had maintenance of reduction at follow-up (less than 1 year).

Much attention has been paid to post-operative care and rehabilitation in the first two cases of ARnF for Broberg & Morrey type II radial head fractures. Continuous passive motion (CPM) was used in the patients after ARnF for one week postoperatively, and then active flexion and extension were encouraged under tolerable pain. All 11 patients in this study acquired functional and painless range of motion within the 6 weeks postoperatively, indicating that stable fixation with ARnF can be maintained until the fractures begin to heal.

No complication was noted in this study for patients underwent ARnF, such result was relatively acceptable when compared to an overall complication rate of 12%, which was reported by Kelly et al. a retrospective review of 473 consecutive elbow arthroscopies performed during an 18-year period (18), with serious complications in less than 1% (permanent nerve lesions or infection), and minor complications (such transient nerve lesions) in 11% of the arthroscopic procedures. Despite the lack of representativeness of the small sample size of the study, the relatively low complication rate in our study may have contributed to the simple procedure and shorter operating time.

To our knowledge, no specific recommendations for using ARnF for Broberg & Morrey type II radial head fractures with mechanical rotation block have been made. Guerra et al. have described in detail the working position and type of fixation for ARIF, and he also demonstrated the indications for ARIF: a. mechanical block in prono-supination movements; b. two-part fractures with displacement greater than 5 mm if involving the head or greater than 4 mm if involving the neck; c. fractures with multiple fragments, but still treated with screw fixation (5). Our treatment algorithm was similar to Guerra's suggestions, but we made a new alternative for treatment after reduction of the fragment and testing the integrity and tension of both the AL and LCL (5). We suggested that ARnF can be used when MCL and LCL keep their integrity and tension for Broberg & Morrey type II radial head fractures (Figure 1). Furthermore, we felt is necessary to list some contraindication for ARnF based on our clinical practice: 1. Active joint infection: An active infection in the affected joint is a contraindication for ARnF, as arthroscopic intervention could worsen an infection or should be deferred until the infection is resolved. 2. Severe joint degeneration or bony ankylosis: If a joint has advanced osteoarthritis, extensive heterotopic ossification, or complete bony ankylosis that prevents adequate arthroscopic access or would not benefit from ARnF, alternative treatments (such as open surgery or arthroplasty) are more appropriate. 3. Underlying medical contraindications to surgery: Patients who cannot undergo arthroscopic surgery safely (e.g., uncorrected coagulopathy or poor general health/anesthesia risk) would also be unsuitable for ARnF. However, the contraindications required further update and modification based on future studies.

Several limitations in the current study are acknowledged. First, the sample size of patients using ARnF was relatively small compared to the sample size of the other real-world studies other studies, as we were limited to the 11 cases meeting inclusion criteria during the study period. Accordingly, we did not perform a prior power analysis; this study was intended as a preliminary exploration of the ARnF technique. Second, the propensity score matching was suboptimal on some covariates, some socioeconomic variables were missing in the disease-specific database including labor work involved, family income, etc. Furthermore, three eligible ARnF patients were excluded due to incomplete data, which may introduce some selection bias. Third, the long-term effects of this technique are unknown; the limited follow-up period of 1 year has implications for the interpretation of the results. Fourth, our cohort consisted of relatively young adults, so the generalizability of this technique to older patients with osteoporotic bone or to more complex radial head fracture patterns is uncertain. Fifth, the variables with some missingness/imprecisions were included in the PSM model, which may lead to substantial bias. Finally, some low-frequency adverse events (e.g., transient nerve palsy, post-procedure joint stiffness requiring additional intervention, or heterotopic ossification) could have been missed due to limited power. Lastly, all ARnF procedures in this study were performed by a single surgeon with extensive elbow arthroscopy experience. While this likely minimized technical variability, it may also limit the generalizability of results to broader clinical settings with varying surgeon expertise. Further randomized controlled trials with larger samples are needed to evaluate ARnF in patients after Broberg & Morrey Type II radial head fractures, future studies should incorporate extended follow-up to assess durability of outcomes and should involve an economic analysis (e.g., cost-effectiveness or cost-benefit evaluation) of the ARnF technique.

## Conclusion

In this initial exploratory study, arthroscopic reduction without fixation (ARnF) for Broberg & Morrey Type II radial head fractures with a mechanical rotation block achieved clinical outcomes comparable to arthroscopic reduction and internal fixation (ARIF). ARnF avoids implant use, thereby reducing implant-related costs and potential complications.

## Data availability statement

The original contributions presented in the study are included in the article/Supplementary Material, further inquiries can be directed to the corresponding authors.
